# Effects of the *skp1* gene of the SCF complex on lipid metabolism and response to abiotic stress in *Chlamydomonas reinhardtii*

**DOI:** 10.3389/fpls.2025.1527439

**Published:** 2025-03-17

**Authors:** Xiao Dong Deng, Meng Wang, Si Hang Liu, Dian Long Xu, Xiao Wen Fei

**Affiliations:** ^1^ Key Laboratory of Tropical Transnational Medicine of Ministry of Education, School of Basic Medicine and Life Sciences, Hainan Medical University, Haikou, China; ^2^ Institute of Tropical Bioscience and Biotechnology, Chinese Academy of Tropical Agricultural Science & Key Laboratory of Biology and Genetic Resources of Tropical Crops of Hainan Province, Hainan Institute for Tropical Agricultural Resources, Haikou, China; ^3^ Hainan Provincial Key Laboratory for Functional Components Research and Utilization of Marine Bio-resources, Haikou, China; ^4^ Zhanjiang Experimental Station, CATAS, Zhanjiang, China

**Keywords:** SCF(SKP1-Cullin1-F-box) complex, Skp1, E3 ubiquitin ligase, Chlamydomonas reinhardtii, lipid metabolism, abiotic stress

## Abstract

SKP1 (S-phase kinase-associated protein 1) is a key member of the SCF (SKP1-Cullin1-F-box) E3 ligase complex. The SCF complex is involved in regulating various levels of plant physiology, including regulation of cellular signaling and response to abiotic stresses. While the function of SKP1 in plants is well known, its function in algae remains poorly understood. In this study, we investigated the role of the *Chlamydomonas reinhardtii skp1* gene using RNAi interference and overexpression approaches. Subcellular localization of SKP1 was performed by transient expression in onion epidermal cells. For abiotic stress assays, the growth of *skp1* RNAi and overexpression recombinant strains was examined under conditions of high osmolality (sorbitol), high salinity (NaCl) and high temperature (37°C). Our results showed that *skp1* silencing significantly reduced oil accumulation by 38%, whereas *skp1 over*expressing led to a 37% increase in oil content, suggesting that *skp1* plays a crucial role in regulating oil synthesis and may influence lipid accumulation by regulating photosynthetic carbon flux partitioning. Subcellular localization analysis revealed that *skp1* was predominantly localized within the nucleus. Furthermore, our results showed that SKP1 responds to abiotic stresses. Under sorbol and NaCl stress conditions, RNAi interference strains exhibited better growth than controls; however, their growth was comparatively impaired under 37°C stress compared to controls. On the other hand, overexpression strains showed weaker growth under sorbol and NaCl stress but were more tolerant to 37°C heat stress. These results illustrate the functional diversity of SKP1 in *Chlamydomonas*. This study provides an important complement for lipid metabolism and abiotic stress regulation in microalgae.

## Introduction

Biodiesel produced from microalgae is considered as a new generation of biofuel. Microalgae have attracted the attention of companies and researchers due to their unique advantages such as high photosynthetic efficiency, effective use of industrial wastewater for production, and less land occupation (Enamala et al., 2018). High cultivation cost is the main obstacle limiting the development of microalgal biodiesel. Therefore, many studies have been conducted to improve the lipid and biomass of microalgae by genetic engineering, hoping to solve the problem of algal species problem at the source. Currently, two triacylglycerol (TAG) synthesis pathways have been identified in microalgae, namely the Kennedy pathway and the phospholipid: diacylglycerol acyltransferase (PDAT) mediated TAG synthesis (Yoon et al., 2012; Wang et al., 2020). In *C. reinhardtii*, key enzymes involved in the Kennedy pathway include diacylglycerol O-acyltransferase (DGAT), glycero-3-phosphate acyltransferase (GPAT), phosphatidic acid phosphohydrolase (PAP) and glycero-3-phosphate dehydrogenase (GPDH) (Driver et al., 2017; Hung et al., 2013; Deng et al., 2013a; Herrera-Valencia et al, 2012). Increasing or inhibiting the expression of these genes has been used to increase the lipid content of microalgae with some success (Driver et al., 2017; Deng et al., 2012; Russa et al., 2012). In addition, enzymes that regulate fatty acid metabolism such as acyl-ACP thioesterase (Fat), acetyl-coA carboxylase (ACCase), and phosphoenolpyruvate carboxylase (PEPC) have also been shown to be involved in TAG biosynthesis (Tan and Lee, 2017; Kao and Ng, 2017; Chen et al., 2019). In recent years, Luo et al. Have also found that ubiquitination in microalgae may be involved in the regulation of lipid metabolism (Luo et al., 2015; Luo et al., 2018; Luo et al., 2021; Luo et al., 2022). Therefore, it is necessary to elucidate the mechanism of lipid biosynthesis in microalgae in order to promote biodiesel production in microalgae.

Ubiquitination is an important protein modification mode for regulating cellular metabolism in eukaryotic cells, which binds ubiquitin to target proteins for degradation. The following components are required for protein ubiquitination in eukaryotic cells: ubiquitin activase (E1), ubiquitin-conjugating enzyme (E2), ubiquitin ligase (E3), proteasome complex (26S) and deubiquitinating enzyme (DUB). Among them, E3 ubiquitin ligase is the main one that binds target proteins (Ebner et al., 2017; Damgaard, 2021). Currently, E3 ubiquitin ligases are classified into two categories: single-subunit and multisubunit. Single-subunit E3 include four types: RING type, U-box type, HECT type (homology to E6-associated carboxy-terminus) and RBR type (ring between ring) (Stone and Callis, 2007). The multi-subunit E3 mainly includes the CRLs family (Cullin-RING ligases family) and the anaphase promoting complex/cyclosome. The family of CRLs in multisubunit E3 can be classified according to the different types of Cullin subunits, and the Cullin family is evolutionarily conserved in different species, e.g., there are seven types of Cullin in human, five types of Cullin in *Arabidopsis*, and three types of Cullin in yeast (Moon et al., 2004). Different types of Cullin subunits bind different substrate recruiting proteins belonging to the F-box, BTB or DDB-binding WD40 (DWD) family (Stone, 2014). One of these CRLs based on the CUL1 type is known as SCF (SKP1-Cullin1-F-box) type E3 (Stone and Callis, 2007; Marín, 2009; Roodbarkelari et al., 2010; Dias et al., 2002; Gray et al., 1999; Seo et al., 2014).

Currently, 21, 31, 19 and 15 *skp1*-like genes have been identified in *Arabidopsis thaliana*, rice, tomato and chickpea, respectively (Zhao et al., 2003; Kong et al., 2007; Zhang et al., 2015; Varshney et al., 2023). *skp1*-like genes have also been found in several species such as wheat *skp1*, soybean *skp1*, chilli *skp1*, citrus *skp1* and peony *skp1* (Chai et al., 2010; Hong et al., 2013; Hao et al., 2017; Chen et al., 2018; Lim et al., 2019). The SCF complex formed by SKP1 is involved in various levels of eukaryotic regulation such as regulation of intracellular hormone signaling, meiosis, flower development, light signaling, seed germination, circadian rhythms, cellular senescence, and biotic and abiotic stress responses (Gray et al., 1999; Zhao et al., 2003; Hao et al., 2017; Chen et al., 2018; Lim et al., 2019; Zou et al., 2020; So et al., 2020; Rao and Virupapuram, 2021; Marrocco et al., 2003; Takahashi et al., 2004; Dezfulian et al., 2012; Kuroda et al., 2012; Yang et al., 1999; Yang et al., 2006; Wang and Yang, 2006; Lu et al., 2016; Xu et al., 2002; Guo and Ecker, 2003; Ni et al., 2004; Parry and Estelle, 2006; Shao et al., 2023).

A large number of studies have shown that stress conditions can induce lipid accumulation in microalgae. These conditions include high temperature and deficiencies of nitrogen, sulfur, phosphorus, iron and silicon (Holcomb et al., 2011; Longworth et al., 2016). Of these, nitrogen deficiency induces the most significant accumulation of neutral lipids. The pathway for lipid synthesis in microalgae is similar to that of higher plants (Bellou et al., 2014), but differs in that there are several bypass pathways for lipid metabolism in algal cells (Bellou et al., 2014; Lee et al., 2017). The discovery of genes involved in the regulation of lipid metabolism is important to unravel the lipid metabolism network of microalgae and promote the production of microalgal biodiesel. Therefore, the digital gene expression profile of *C. reinhardtii* under nitrogen stress was used to screen genes associated with the regulation of lipid metabolism in our laboratory. It was observed that *skp1* RNA abundance increased significantly under nitrogen-deficient culture (Li et al., 2012a), which is concomitant with increased lipid accumulation in *C. reinhardtii* under nitrogen stress. To investigate the relationship between abnormal *skp1* gene expression and lipid accumulation, this study used RNAi interference and overexpression technology to explore the mechanism by which *skp1* regulates changes in *Chlamydomonas* lipids. In addition, this study analyzed the effects of high osmolarity (sorbol), high salt and high temperature conditions on *skp1* gene expression, thus providing a scientific basis for the genetic modification of microalgae in favor of lipid and biomass increase.

## Materials and methods

### Bioinformatics analysis of protein homologs

To identify *skp1* genes in *Chlamydomonas* and other organisms, the SKP1 domain of the *Arabidopsis* ASK protein was used as a seed sequence to search the Phytozome (http://www.phytozome.net/), Plaza (http://bioinformatics.psb.ugent.be/plaza/) and NCBI (National Center for Biotechnology Information) databases through BLASTP. Online MEME (https://meme-suite.org/meme/tools/meme) was used to predict the conserved motifs of *skp1* genes, and an online InterProScan analysis (http://www.ebi.ac.uk/InterProScan) was carried out to identify the domains of the SKP1 proteins (Jones et al., 2014). A phylogenetic tree was then constructed using MEGA11 software based on the alignment of SKP1 protein homologs in *Chlamydomonas* and other organisms. The tree was generated using the neighbor-joining (NJ) method with Poisson correction and pairwise deletion parameters. Based on the sequence information obtained from the online databases, the schematic representation of the SKP1 homolog structures was created.

### Algal strain, cultivation conditions, and biomass assay

C. *reinhardtii* CC425 was obtained from the Institute of Hydrobiology, Chinese Academy of Sciences. Cells were grown on a tris-acetate-phosphate (TAP) agar plate and inoculated into 100 mL Erlenmeyer flasks containing 50 mL of TAP, N-deficient TAP (TAP-N) and S-deficient TAP (TAP-S)media (Harris, 1989). The TAP-N medium contained the same components but with NaCl instead of NH_4_Cl, while TAP-S medium contained the same components but with NaCl instead of NH_4_SO_4_. All cultures were maintained in an incubator shaker (180 rpm at 25°C) and received continuous light from normal fluorescent lamps with a light intensity of 150 μmol·m-2·s-1. The cultured *Chlamydomonas* cells were centrifuged at 5000 rpm for 5 min, and the precipitate was collected after removing the supernatant. The algal cells were processed through a vacuum centrifugal dryer (8000 rpm,0.5 mbar,1 hour) to obtain the dried cells. Biomass concentration (g/L) was determined by measuring the OD490 value as described in a previous study (Deng et al., 2011; Deng et al., 2013b). To generate the standard curve, a series of *C. reinhardtii* CC425 samples with different biomass concentrations were collected. The OD490 and cell dry weight were determined gravimetrically on the dried cells, and the standard curve of OD490 versus biomass concentration (g/L) was plotted. Samples were diluted in appropriate ratios to ensure that the measured OD490 values ranged from 0.04 to 1. Biomass concentration was then calculated using the following formula: Biomass concentration(g/L) = 0.7444 × OD490 – 0.0132 (**Supplementary Figure 4**).

### Analysis of lipid content

The Nile Red fluorescence method was used to determine lipid levels, as described in previous studies (Gao et al., 2008; Huang et al., 2009; Chen et al., 2009; Deng et al., 2011; Deng et al., 2013b). Algal cells were directly stained with 0.1 μg/mL Nile Red for 10 min, and fluorescence was measured using a GloMax^®^-Multi Detection System (Promega, USA) with excitation and emission wavelengths of 470 nm and 570 nm, respectively. The fluorescence value was calculated by the equation: FD (470/570) = (A2−A1), where A2 is the fluorescence value of algal cells after staining with Nile Red, A1 is the fluorescence value of algal cells before staining. To establish the relationship between the fluorescence value of the samples and their neutral lipid content, a standard curve was drawn by preparing different concentrations of Triolein (Sigma, USA) and determining their fluorescence value after staining with Nile Red. The lipid content of the algal cells was then calculated using the following formula: Lipid content (g/g) = [0.0004 × FD (470/570) − 0.0038] × 0.05/cell dry weight (**Supplementary Figure 5**). Images for the microscopic assay were captured using a fluorescence microscope after Nile Red staining. After staining the cells with Nile Red (10 μg/mL final concentration), images were acquired using a Nikon 80i Fluorescence Microscopes. Nile Red signals were captured with an excitation wavelength of 480 nm, and emission was collected between 560 nm and 600 nm (Huang et al., 2009; Chen et al., 2009).

### Total RNA extraction

Total RNA was prepared according to the method reported by Li et al (2012b) with modifications. Cells from cultured algae were collected by centrifugation at 10,000g for 1 min. After a series of phenol/chloroform extractions, nucleic acids were precipitated with 100% ethanol and washed with 75% ethanol. Finally, the dry pellet was dissolved in 50μL of RNase-free water. RNA concentration was quantified by spectrophotometry and integrity was assessed by agarose gel electrophoresis.

### Cloning of the *skp1* gene and generation of an overexpression vector

The *Chlamydomonas skp1* gene, identified in the Phytozome database as Cre12.g501200. First-strand cDNA was synthesized using the SuperScript III Reverse Transcriptase Kit (Invitrogen, USA). A fragment of the *skp1* gene was amplified by PCR using the primers skp1F: 5’ -CGCTCTACACAAATCGCAAC-3’ and skp1R: 5’-ATCAGACCCGTGCTTAATCG-3’. After purification using a DNA gel extraction kit (TaKaRa, Japan), the DNA was inserted into the vector pMD18-T (TaKaRa, Japan) and the resulting plasmid was designated pMD18T-skp1. The sequence of the cloned *skp1* gene was verified by sequencing. In addition, the *skp1* gene was amplified using primers 5’-GAAGATCTGGCCACCAAGGTGAAGCTTA-3’ and 5’-GGACTAGTTTAATCGAAAGCCCACTGGT-3’ with the addition of Bgl II and Spe I restriction sites added. The resulting product was digested with Bgl II and Spe I and then cloned into the expression vector pCAMBIA1302. After PCR verification, the plasmid was designated pCAM-skp1.

### Construction of the RNAi vector against *skp1* gene

To construct the RNAi vector against the *skp1* gene, the fragment of *skp1* and its reverse complementary sequences were amplified by PCR using *C. reinhardtii cDNA* as the template and the primers skp1RNAiF: 5′-TCGATTAAGCACGGGTCTGA-3′ and skp1RNAiR: 5′-CAAATCCTCCCTACCGCCAC-3′.The PCR fragment was then digested with Hind III/BamHI and XbaI/SalIand then inserted into the appropriate cloning site of an intermediate vector to obtain pMD18-skp1F-18S-skp1R containing the skp1 inverted repeat sequence (skp1 IR) and finally the skp1 IR was inserted into the EcoR I digested pMaa7IR/XIR to generate pMaa7IR/skp1IR.

### Transformation of *Chlamydomonas*

Transformation of *C. reinhardtii* strain CC425 was performed as described by Kindle (1990). *C. reinhardtii* cells were grown in TAP medium until reaching a density of 2×10^6^ cells/mL. Cells were harvested by centrifugation at 4000 rpm for 3 min, washed twice and resuspended in TAP medium to reach a cell density of approximately 1×10^8^/mL. Plasmid DNA was transformed into the cells by the glass bead method. For each experiment, 2 μg of plasmid DNA was mixed with 400 μL of cells, 100 μL of 20% polyethylene glycol, and 300 mg of sterile glass beads, and the reaction mixture was vortexed for 15 seconds to allow the plasmid to enter the algal cells. To induce RNAi or gene expression, cells were allowed to recover for one day before plating onto selective media. RNAi transformants were selected on TAP medium containing 1.5 mM L-tryptophan, 5μg/mL paromomycin, and 5μM 5-FI. Meanwhile, pCAM-skp1 transformants were selected on TAP medium with 50μg/mL hygromycin. Cells on the plates were grown under low light (approximately 50 μmol·m-2·s-1 photosynthetically active radiation). After 6-7 days, the algal colonies that appeared on the plate were picked and inoculated into another plate with the same antibiotic. After new algal colonies had grown, more than 10 transformed strains were randomly selected and inoculated into 30 ml of TAP liquid medium for shaking culture for 2-3 days. The algae were collected by centrifugation and the DNA of each transformed strain was extracted using the TaKaRa MiniBEST Universal Genomic DNA Extraction Kit(Takara, Japan). The positive transformed algal strains were identified by PCR using this DNA as a template and the primers listed in **Supplementary Table 2**.

### Quantitative PCR

Real-time PCR analysis was performed as previously described by Deng et al. (2011).RNA was isolated using the TRIzol kit (Takara, Japan). cDNA was synthesized using an Invitrogen SuperScript cDNA Synthesis kit(Invitrogen, USA). Real-time PCR was performed using SYBR Green as the fluorescent dye. 18S rRNA was used as an internal control, using the primers 18S rRNAF (5’-TCAACTTTCGATGGTAGGATAGTG-3’) and 18S rRNAR (5’-CCGTGTCAGGATTGGGTAATTT-3’). Gene-specific primers (**Supplementary Table 2**) were used to determine the amount of target cDNA. The transcript’s amplification rate (Ct) of each transcript was estimated using the PCR baseline subtraction method in the iCycler program at a constant fluorescence level. Ct values were determined over three replicates. Relative fold differences were calculated using the relative quantification analytical approach (2-^△△Ct^) (Livak and Schmittgen, 2001).

### Construction and subcellular localization of the *skp1*-GFP fusion expression vector

Using the plasmid containing the full-length *skp1* gene as a template, we designed primers with *Bgl* II and *Spe* I sites at either end: 5’- GAAGATCTGGCCACCAAGGTGAAGCTTA -3’ and 5’- GGACTAGTATCGAAAGCCCACTGGTTCT -3’. The full-length *skp1* gene (with the termination codon removed) was amplified by PCR using these primers. The PCR product was then digested with *Bgl* II and *Spe* I restriction enzymes, ligated into pCAMBIA1302 vector, and transformed into *E. coli* DH5αto generate the skp1-GFP fusion expression vector. Onion epidermis transformation was performed using a gene gun method (Yu et al., 2014). After one day of dark incubation, the expression of skp1-GFP in transformed onion cells was observed under a laser confocal microscope using blue light (488 nm excitation).

### Abiotic stress test

The *skp1* RNAi interference strain and the *skp1* overexpression strain were inoculated into 50mL of TAP medium for shaking culture. When the cell density reached the mid-logarithmic growth stage (2x10^6^ cells/mL), they were centrifuged and washed twice with the same medium, and finally resuspended in 5mL of fresh medium. The cell concentration was determined by calculating the cell number as described by Harris, prior to upsampling and sample gradient dilution [66]. After gradient dilution, 5 μL samples were spotted on plates with different treatments (TAP, TAP+300 mM sorbol, TAP+170 mM NaCl, and TAP incubated at 37°C) with 12,000, 6,000, 3,000, 1,500, and 750 cells, respectively. The results were observed after incubation at 25°C for 2-3 days. Similarly, 500 μL of algal solution with the same concentration was inoculated into TAP liquid medium containing different concentrations of NaCl (100 mM NaCl, 170 mM NaCl, 300 mM NaCl) and 300 mM sorbol, respectively. The growth conditions of the algal cells were assessed and recorded. The experiment was repeated three times.

### Statistical analyses

SPSS 25.0 was used to analyze the data. Duncan’s multiple range test and Student’s t-test were used to examine significant differences between means. Numerical results are presented as mean ± standard deviation with 99% confidence intervals and p < 0.01 was considered statistically significant.

## Results

### SKP1 proteins

A large collection of amino acid sequences was retrieved through Blast searches, and a selection of SKP1 homologs (**Supplementary Table 1**) was chosen for protein domain and phylogenetic analyses. No SKP1 homolog was found in *Eubacteria* or *Archaea*. However, a single SKP1 homolog was identified in each of the algae, protists, fungi, humans, and other vertebrates, while species from monocotyledons, dicotyledons, arthropods, and nematodes contain multiple *skp1* genes. To determine the evolutionary relationship between *Chlamydomonas* and its counterparts in other organisms, a phylogenetic analysis of SKP1 protein sequence was performed. The unrooted phylogenetic tree was constructed based on the full-length SKP1 protein sequences from species across vertebrates, arthropods, platyhelminthes, echinoderms, nematodes, protists, green algae, mosses, ferns, fungi, gymnosperms, monocots, and eudicots. As shown in **Supplementary Figure 1**, *Chlamydomonas skp1* is genetically close to the green algae *Tetradesmus obliquus* and *Scenedesmus* sp. It is also grouped in a large branch with the bryophytes *Physcomitrium patens* and *Marchantia polymorpha*, the gymnosperm *Pinus taeda*, and the fern *Ceratopteris richardii*. An online Interproscan analysis (http://www.ebi.ac.uk/InterProScan) was performed to identify the conserved domains within each SKP1 protein. As shown in **Supplementary Figure 1**, all the SKP1 proteins contain both IPR016072 and IPR016073, which are located at the C-terminal and N-terminal regions of each SKP1 protein, respectively.

### Expression analysis of *skp1* under nitrogen-deficient cultivation of *Chlamydomonas*

The *skp1* mRNA level showed a continuous increase as the cultivation time of *C.reinhardtii* CC425 was prolonged under -N conditions. Compared to the control group (normal N), there was a significant increase in the *skp1* mRNA level under N deficient conditions (**Supplementary Figure 2**), indicating that *skp1* responds to low nitrogen stress.

### Transformation of the *skp1* RNAi and overexpression vector into *Chlamydomonas*

**Figure 1:**
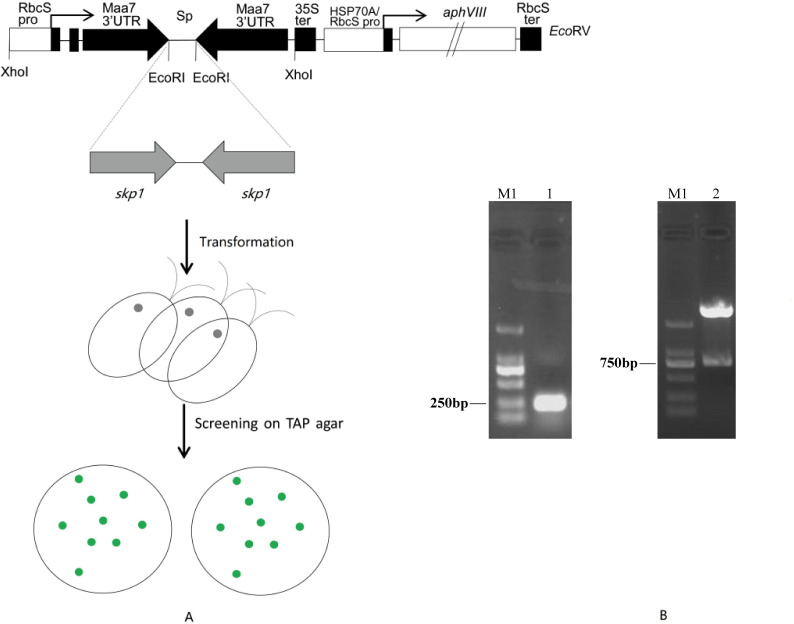
**(A)** Co-silencing of tryptophan synthase β subunit (Maa7) and the target gene skp1 induced by the expression of the tandem Maa7/skp1 inverted repeat (IR) transgenes. A diagram of the construct used to drive RNAi, generated by modifying the ble-Maa7 IR transgene. Fragments corresponding to the target gene were cloned in both sense and antisense orientations, flanking a DNA spacer (Sp), within the arms of the Maa7 3’UTR IR. The Maa7 IR/skp1 IR transgene is controlled by the RbcS2 promoter (RbcS pro) and the Cauliflower Mosaic Virus 35S terminator (35S ter). This construct is designed to produce, upon transcription, an RNA that folds into a hairpin loop structure with a stem of tandem IRs. The previously engineered aminoglycoside 3’-phosphotransferase (aphVIII) gene was placed downstream of the Maa7 IR/skp1 IR transcription unit. **(B)** PCR amplification and EcoRI digestion analysis of the skp1 interference fragments. M1, DNA ladder 2000; lane1, skp1 PCR amplification fragments (275bp); lane2, recombinant plasmid pMaa7IR/skp1IR digested with EcoRI.

Using cDNA synthesized from *Chlamydomonas* total RNA as a template, we successfully amplified the *skp1* RNAi interference fragment (**Figure 1B**, **Supplementary Figure 3**) with an expected size of 275bp. After cloning, the *skp1* fragment was determined by sequencing analysis with a 100% homology to the *skp1* gene sequence in the Phytozome database. The forward and reverse fragments of the *skp1* gene were recovered and then inserted into the RNAi vector of pMaa7 IR/XIR (**Figure 1A**). Single clones were selected and identified by *EcoR*Idigestion of the recombinant plasmids (**Figure 1B**, **Supplementary Figure 3**), resulting in the generation of the *skp1* RNAi interference vector pMaa7IR/skp1IR. The full-length s*kp1* gene DNA was amplified and subsequently cloned into the pCAMBIA1302 expression vector. The recombinant plasmid, pCAM-skp1, was confirmed by PCR amplification (510 bp) (**Supplementary Figure 6A**). After transformation of *C. reinhardtii* CC425 with the RNAi vector, 17 positive recombinant algal clones were identified by PCR, of which three were randomly selected for further experiments. Meanwhile, after transformation of *C. reinhardtii* CC425 with the overexpression vector, 25 positive recombinant algal clones were identified by PCR, three of which were randomly selected for subsequent experiments (**Supplementary Figures 6B, 6C**).

### *skp1* positively regulates lipid accumulation

**Figure 2:**
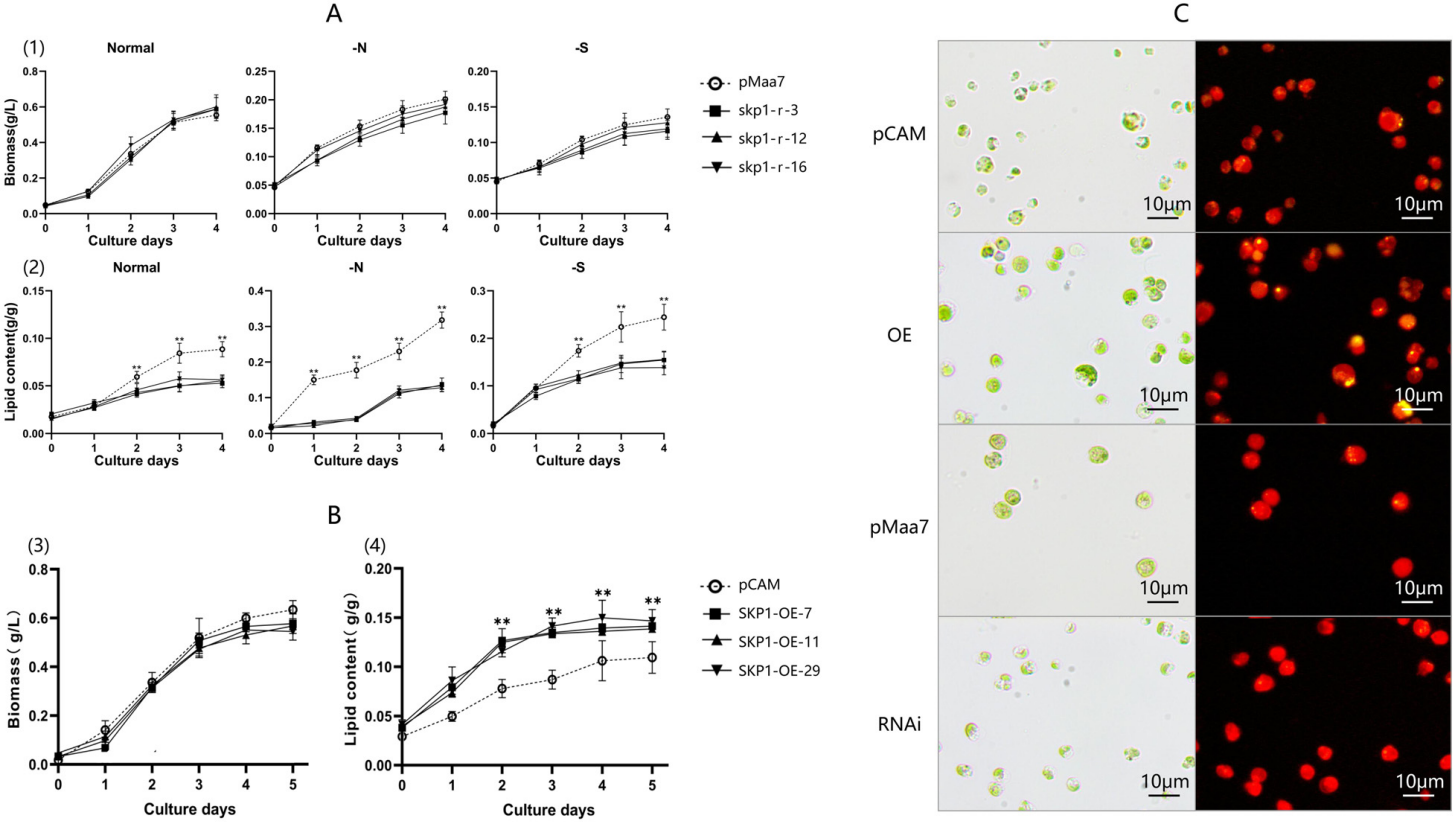
Biomass and lipid content analysis of RNAi and overexpression recombinant strains. **(A)**, Biomass accumulation and lipid content of skp1 RNAi recombinant strains under normal, -N, and -S conditions. (1), Biomass accumulation of skp1 RNAi recombinant algal strains under normal, -N, and -S conditions. (2), Lipid content of skp1 RNAi recombinant algal strains under normal, -N, and -S conditions. **(B)** Biomass accumulation and lipid content of skp1 overexpression recombinant strains. (3), Biomass accumulation of skp1 overexpression recombinant algal strains. (4), Lipid content of skp1 overexpression recombinant algal strains. **(C)** Microscopic observation of skp1 RNAi and overexpression transformants. The yellow areas represent oil droplets. The oil droplets in the skp1 interference algal strains were significantly fewer than those in the control (Maa7IR/XIR transformed algal strains), indicating that the interference with the skp1 gene significantly inhibits oil accumulation in the algal cells. pMaa7, control, algal strain transformed with the empty pMaa7IR/XIR plasmid; skp1-r-3, skp1-r-12 and skp1-r-16, skp1 RNAi recombinant algal strains. pCAM, control, algal strain transformed with the pCAMBIA1302 plasmid; SKP1-OE-7,SKP1-OE-11 and SKP1-OE-29, skp1 overexpression recombinant algal strains. OE, recombinant algal strain transformed with pCAM-skp1 (skp1 overexpression recombinant strain 29); RNAi, recombinant algal strain transformed with pMaa7IR/skp1IR (skp1 RNAi recombinant strain 16).**, P<0.01.

The selected positive transformed strains were then analyzed for biomass, oil content, and *skp1* gene mRNA level. The results showed a progressive increase in biomass for all skp1 RNAi recombinant strains over time, with no significant difference observed in the growth curve of *skp1* RNAi interference strains compared to the control under normal, nitrogen deficient, and sulfur deficient conditions. These results suggest that *skp1* interference has no effect on biomass changes (**Figure 2A**). Analysis of lipid content showed a 38% decrease in *skp1* RNAi recombinant strains compared to the control under normal culture conditions, a 58% decrease under nitrogen deficient conditions and a 39% decrease under sulfur deficient conditions. This indicates the interference with *skp1* significantly inhibits lipid accumulation. Furthermore, lipid accumulation was significantly higher under nitrogen deficient and sulfur-deficient conditions compared to the control, with nitrogen deficiency leading to the highest increase in oil content (**Figure 2A**).

In contrast, each *skp1* overexpression transgenic algal strain showed an increase in biomass over time, with no significant difference observed between the growth curve of the *skp1* overexpression transgenic strains and the control strain (**Figure 2B**). Analysis of lipid content in the *skp1* overexpression strain revealed a 37% higher lipid content compared to the control under normal culture conditions, indicating that *skp1* overexpression significantly promotes lipid accumulation (**Figure 2B**).

Microscopic observations also revealed a reduced lipid content in the *skp1* RNAi interference algal strain, as evidenced by fewer oil droplets compared to the control strain (pMaa7IR/XIR transformed algae strain), where almost no oil droplets were observed (**Figure 2C**). In contrast, microscopy observations showed an increased lipid content in the *skp1* overexpression strain compared to the control (the pCAMBIA1302 transformed strain) (**Figure 2C**). These results suggest that interference with *skp1* significantly inhibits intracellular lipid accumulation, while overexpression of the *skp1* gene greatly enhances lipid accumulation

**Figure 3:**
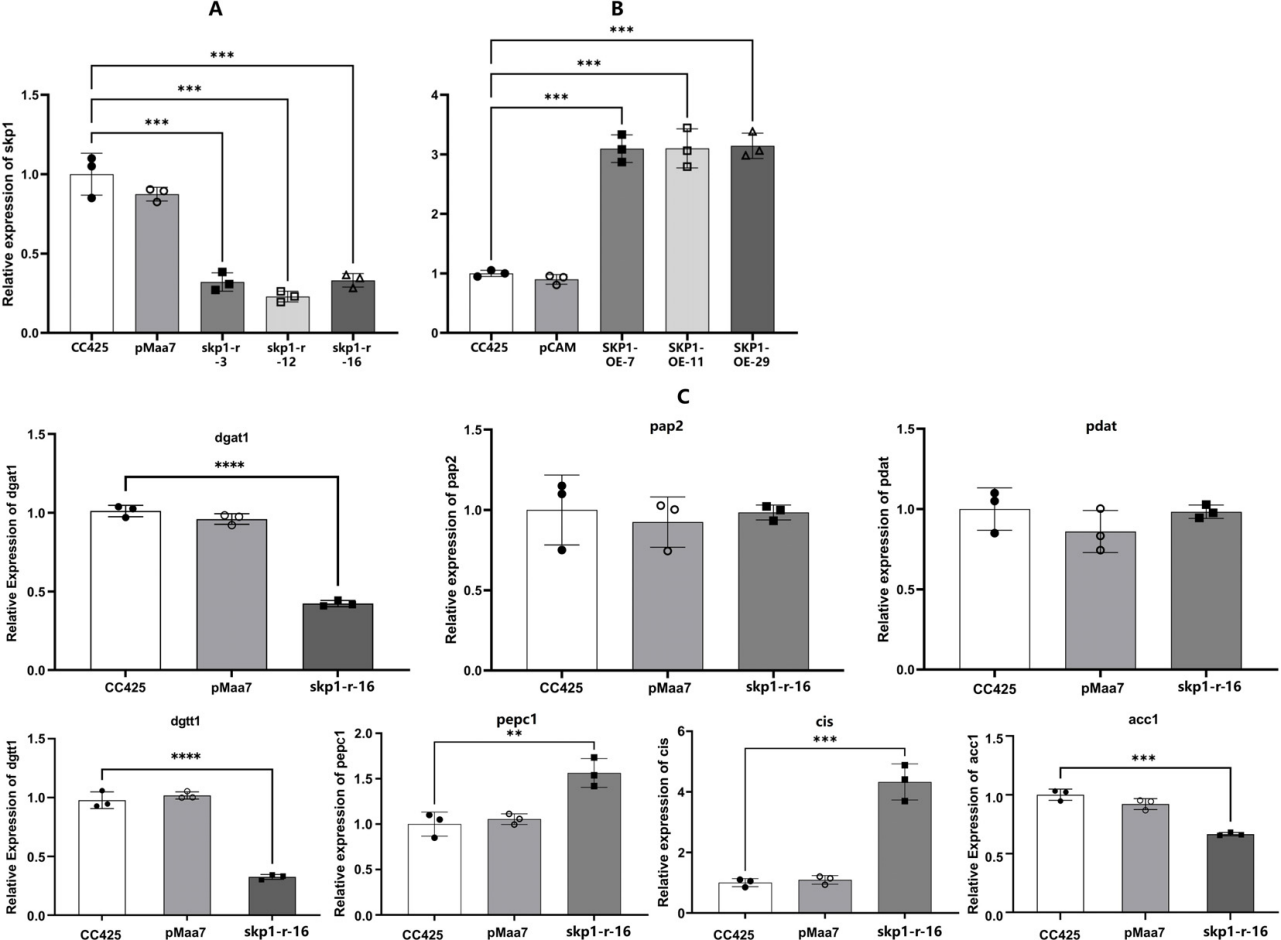
mRNA levels in recombinant algal strains of skp1 and potentially critical genes of the lipid metabolism pathway in C. reinhardtii. **(A)**, skp1 mRNA levels in algal strains with skp1 RNAi disruption; **(B)**, skp1 mRNA levels in skp1 overexpressed algal strains; **(C)**, mRNA levels of pap2, pdat3, pepc1, and cis genes in skp1 RNAi recombinant algal strain. CC425, wild-type Chlamydomonas; pMaa7, algal strain transformed with pMaa7IR/XIR; pCAM, algal strain transformed with pCAMBIA1302;skp1-r-3,skp1-r-12 and skp1-r-16: skp1 RNAi recombinant algal strains; SKP1-OE-7,SKP1-OE-11 and SKP1-OE-29, skp1 overexpression recombinant algal strains. **, P<0.01, ***,P<0.001.

The expression of the target gene in the *skp1* RNAi interference strains and overexpression recombinant strains was assessed using real-time PCR to determine the abundance of *skp1* mRNA on the fourth day of culture. Compared to control strains *C. reinhardtii* CC425 and pMaa7 IR/XIR transgenic algal strains, a significant reduction (70-75%) in *skp1* mRNA abundance was observed in the *skp1* RNAi interference algal strain (**Figure 3A**). Conversely, *skp1* mRNA abundance increased by 225% in the overexpression recombinant algal strains compared to both the control wild strain *C. reinhardtii* CC425 and the pCAMBIA1302 transgenic algal strain (**Figure 3B**). These results indicated that the *skp1* gene was effectively silenced in the *skp1* RNAi recombinant algal strains, while its expression was significantly increased in the overexpress recombinant algal strains.

Moreover, we examined the expression of critical genes in the *C. reinhardtii*’s lipid metabolism pathway, such as *dgat1*(diacylglycerol O-acyltransferase, type 1), *dgtt1*(diacylglycerol O-acyltransferase, type 2), *pap2* (type-2 phosphatidic acid phosphatase), and *pdat*(phospholipid diacylglycerol acyltransferase), as well as *pepc1*,*cis*(citrate synthase) and *acc1*(carboxyltransferase), which influence the direction of carbon flow in *C. reinhardtii* cells. It was found that the expression of the *pap2* and *pdat3* genes was not significantly altered in the *skp1* RNAi recombinant algal strain. However, the expression of *dgat1* and *dgtt1* genes, which affect lipid synthesis, decreased significantly. While the expression of *acc1*, which affects the direction of photosynthetic carbon flow, decreased significantly, the expression of *cis* and *pepc1* increased significantly (**Figure 3C**), implying that the photosynthetic carbon mainly flowed to the tricarboxylic acid cycle, and the carbon flow to fatty acid synthesis was reduced.

### Subcellular localization of SKP1

**Figure 4:**
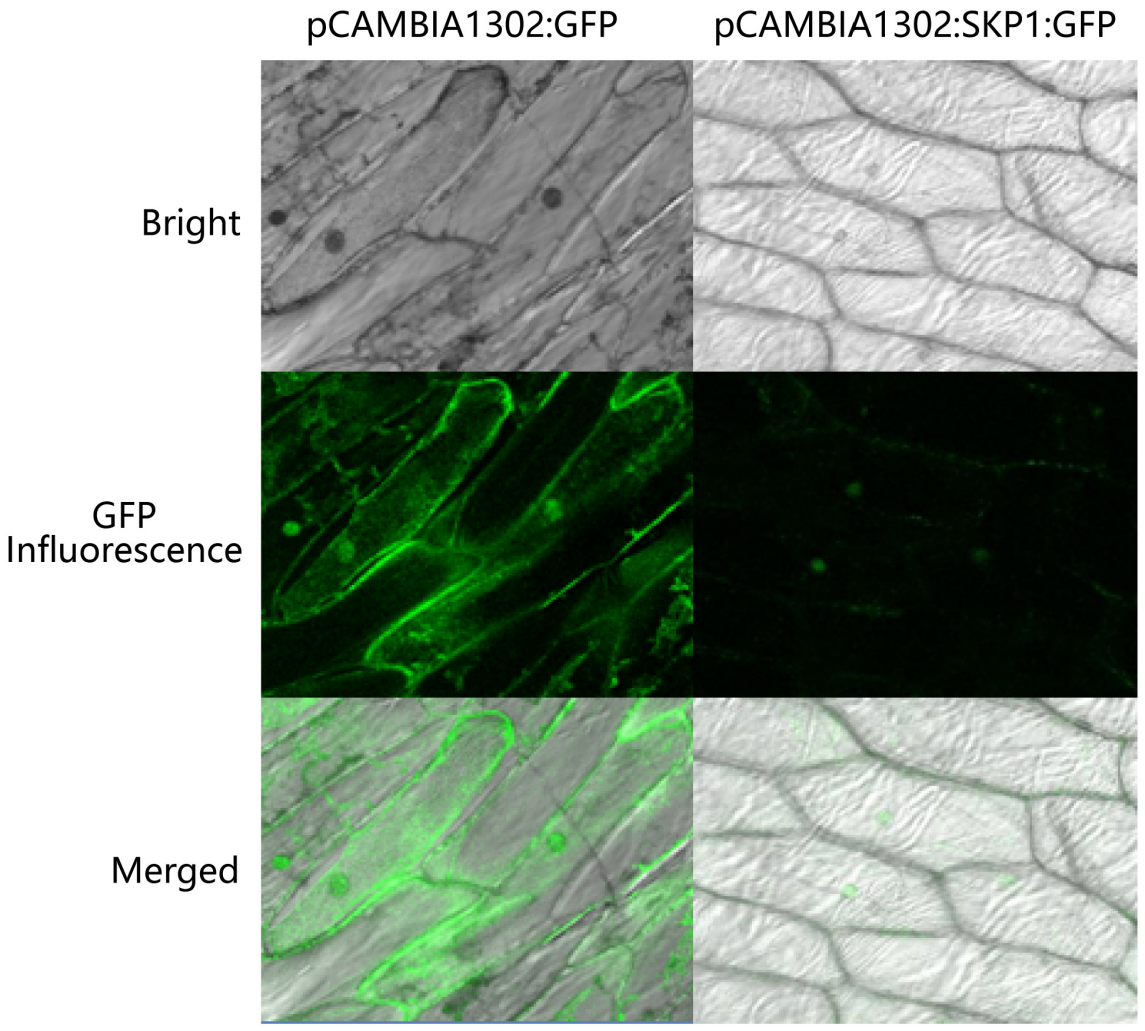
Localization of skp1 in onion epidermal cells. pCAMBIA1302:GFP, pCAMBIA1302 transformed algal strain; pCAMBIA1302:SKP1:GFP, pCAMBIA1302 with skp1:GFP fusion protein transformed algal strain.

To investigate the subcellular localization of skp1, the constructed skp1-gfp fusion expression vector was introduced into onion (*Allium cepa*) epidermal cells using a gene gun method. The empty vector transformant pCAMBIA1302 was used as a positive control (*gfp* gene). The results showed that *gfp* expression was observed in both the cytoplasm and the nucleus of the positive control, indicating that the 35S CaMV promoter effectively drove *gfp* gene expression. In onion cells transformed with skp1-gfp by gene gun delivery, fluorescence signals were exclusively localized to the nucleus and not observed in other cellular compartments or at the cell membrane surface, suggesting that SKP1 is predominantly localized to the nucleus where it exerts important regulatory functions (**Figure 4**).

### Abiotic stress test

**Figure 5:**
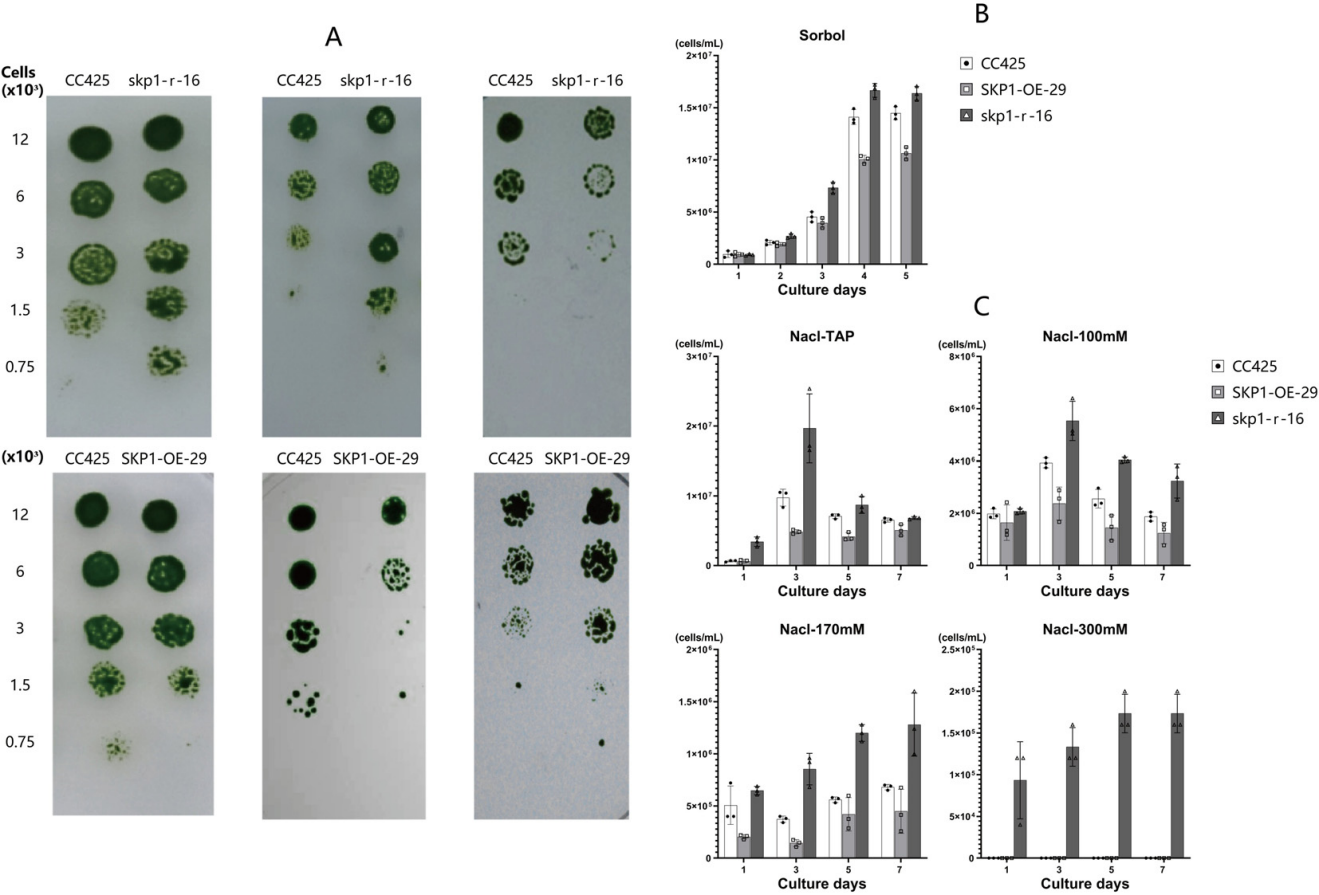
Abiotic stress analysis of skp1 RNAi and overexpression of recombinant algal strains. **(A)** Growth and survival of RNAi (up) and overexpression (down) recombinant strains on plates containing TAP supplemented with sorbol (300mM), NaCl (170mM), and incubated at 37°C. **(B)**, Biomass of skp1 RNAi interference and skp1 overexpressing algal strains in medium supplemented with 300mM sorbol. **(C)**, Biomass of skp1 RNAi interference and skp1 overexpressing algal strains in media supplemented with 100mM, 170mM, and 300mM NaCl. CC425, wild type Chlamydomonas; skp1-r-16, skp1 RNAi recombinant algal strain 16; SKP1-OE-29, skp1 overexpression recombinant algal strain 29.

Growth of the *skp1* RNAi interference and *skp1* overexpression algal strains on plates treated with different stresses showed that, compared to the control (*C. reinhardtii* CC425), the *skp1* RNAi interference strain (skp1-r-16) exhibited enhanced growth under sorbol and NaCl stress, but was less robust than the control under 37°C stress (**Figure 5A**). Conversely, the *skp1* overexpression strain (SKP1-OE-29) showed weaker growth than the control under sorbol and NaCl stress, but was more robust than the control under 37°C stress (**Figure 5A**). Algal cell density was determined by shaking flask cultivation for quantification. The results showed that in TAP medium supplemented with 300mM sorbol, the cell density of the *skp1* RNAi interference strain (skp1-r-16) was significantly higher than that of the *C. reinhardtii* control from day 3 onwards, while that of the *skp1* overexpression strain (SKP1-OE-29) was also significantly lower than that of the *C. reinhardtii* CC425 control from day 3 onwards (**Figure 5B**). In TAP medium supplemented with 100mM NaCl, the cell density of the *skp1* RNAi interference strain (skp1-r-16) exceeded that of the *C. reinhardtii* CC425 control from day 3 to day 7. Similarly, in TAP medium supplemented with 170mM NaCl or 300mM NaCl, the cell density of the *skp1* RNAi interference strain (skp1-r-16) exceeded that of the *C. reinhardtii* CC425 control from day 1 to day 7 (**Figure 5C**). On days 3 to 7, when either a concentration of 100mM or 170mM NaCl was added to the TAP medium, the cell density of the *skp1* overexpression strain (SKP1-OE-29) was lower than that of the *C. reinhardtii* CC425 control(**Figure 5C**).

**Figure 6:**
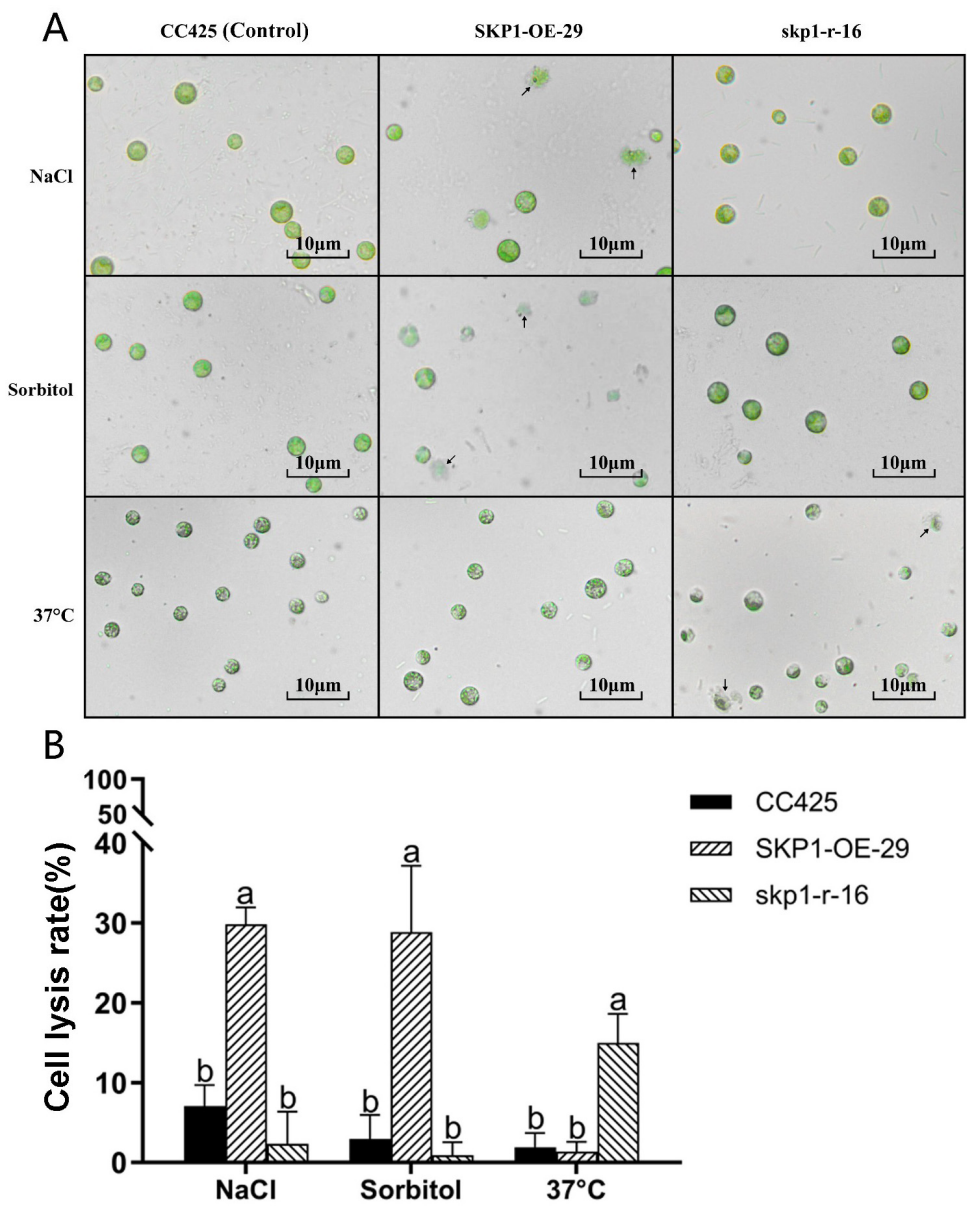
Microscopic observation of algal cell growth under different stress treatments **(A)**, and the corresponding cell lysis rates **(B)**. **(A)** Under NaCl stress, the cells of the skp1 RNAi strain (skp1-r-16) showed robust growth, while partial cleavage was observed in the cells of the skp1 overexpression strain (SKP1-OE-29, arrow pointing to the cleavage cell). Under sorbol stress (high osmolarity), the cells of the skp1 RNAi strains showed robust growth, whereas partial cleavage occurred in the cells of the skp1 overexpression strain (arrow pointing to the cleavage cell). Under high temperature stress (37°C), the cells of the skp1 overexpression strain showed strong growth, while partial cleavage was observed in the cells of the skp1 RNAi strain (arrow pointing to the cleavage cell). **(B)** Analysis of lysis rate of algal cells under different stress treatments. The results showed that the cell lysis rate of skp1 overexpression strain (SKP1-OE-29) was significantly increased under NaCl2 or sorbol stress, and the cell lysis rate of skp1 RNAi strain (skp1-r-16) was also significantly increased under high temperature stress (37°C). CC425, control, wild type Chlamydomonas; skp1-r-16, skp1 RNAi recombinant strain 16; SKP1-OE-29, skp1 overexpression recombinant strain 29. NaCl, 170 mM NaCl + TAP; Sorbol, 300 mM sorbol + TAP; 37°C, algal cells were cultured in TAP at 37°C. Data presented are mean ±SD. Significant differences (p < 0.01) are indicated by different letters.

The above experimental results showed that SKP1 was negative regulation of high osmolarity (sorbol) and salt stress, and positive regulation of high temperature stress. The above conclusions were further validated by microscopic examination of algal cells subjected to different stress conditions. Under NaCl stress, the *skp1* RNAi interference strain (skp1-r-16) showed robust cell growth, whereas partial cleavage was observed in the *skp1* overexpression strain (SKP1-OE-29). Similarly, under sorbol stress (high osmolarity), the *skp1* RNAi interference strain (skp1-r-16) showed healthy cell growth, whereas partial cleavage occurred in the *skp1* overexpression strain (SKP1-OE-29). Conversely, under high temperature stress (37°C), the *skp1* overexpression strain showed vigorous cell growth with no evidence of cleavage compared to the *skp1* RNAi interference strain (**Figures 6A, B**).

## Discussion

Among the E3 ligases, the SCF (SKP1-cullin1-F-box) complex is better understood. It consists of SKP1, Cullin1 and F-box proteins. Dysfunction of any of the core members of the complex can lead to severe defects in various developmental processes of organisms. Among the members of the SCF complex, the F-box protein confers substrate specificity to the complex. Evidence from plants suggests that the SCF (SKP1-cullin1-F-box) complex is involved in a wide range of cellular regulation, including abiotic stress regulation. A search of the F-box proteins in the *C. reinhardtii* genome database (https://phytozome-next.jgi.doe.gov/) revealed that *C. reinhardtii* has a total of 120 F-box or F-box-like proteins, although it contains only one *skp1* gene (**Supplementary Figure 1**). This is similar to the discovery of only one *skp1* gene in yeast, humans and vertebrates (Kong et al., 2004). Phylogenetic analyses of SKP1s confirmed that the SKP1 homologs in vertebrates, invertebrates, fungi, lower plants, and higher plants each form separate monophyletic groups. This suggests that the five major groups indicated in the analyses may indeed reflect the evolutionary history of the SKP1 gene family at very basal levels (**Supplementary Figure 1**). Kong et al. (2004) concluded that the ancestors of eukaryotes as well as those of the protist, fungal, animal, and plant lineages, each shared as few as one SKP1 gene. The protist, fungal, and vertebrate species consistently have one copy of the SKP1 gene, suggesting that the size of the gene family did not increase in these groups. The conservation of a single *skp1* gene through long periods of evolutionary history suggests that either (1) these lineages did not experience gene duplications, or (2) all but one SKP1 homologs have been lost in vertebrates, fungi, green algae, and protists.

As we know, there has been some progress in the study of SKP1 proteins in higher plants. Of the 21 SKP1-like proteins found in *Arabidopsis*, ASK1 and ASK2 have been studied more closely (Liu et al., 2004). ASK1 was found to be involved in *Arabidopsis* meiosis, flower development, hormone and light signaling, and seed germination regulation (So et al., 2020; Rao and Virupapuram, 2021; Marrocco et al., 2003; Takahashi et al., 2004; Dezfulian et al., 2012; Kuroda et al., 2012; Yang et al., 1999; Yang et al., 2006; Wang and Yang, 2006; Lu et al., 2016; Xu et al., 2002; Guo and Ecker, 2003; Ni et al., 2004), as well as in response to abiotic stresses (Varshney et al., 2023; Hao et al., 2017; Chen et al., 2018; Lim et al., 2019). For example, Liu et al. (2015) found that high alkali stress resulted in a significant and rapid induction of *gsskp21* expression, while *gsskp21* overexpression enhanced the tolerance to alkali stress and reduced the sensitivity to ABA by altering the expression of ABA signaling related genes. In other plants, such as peony, wheat, chickpea, soybean, and tomato, SKP1 proteins also play the role in abiotic stress tolerance (Zhang et al., 2015; Varshney et al., 2023; Hong et al., 2013; Hao et al., 2017; Chen et al., 2018). Studies have also implicated SKP1 in the response to biotic stress in plants. For example, in rice, the P7-2 protein encoded by rice black streak dwarf virus can interact with the core subunit of the SCF complex, SKP1 (Tao et al., 2017; Qian et al., 2013). In tobacco, Begomovirus virulence factor βc1 affects tobacco defense against whiteflies by interacting with tobacco SKP1 (Zou et al., 2020). In addition, *Arabidopsis* ASK13 was recently found to bind not only to F-box proteins but also to interact with several other proteins, none of which are components of the SCF complex. This suggests that ASK13 may be involved in a variety of cellular processes in *Arabidopsis* other than ubiquitination (Rao et al., 2018). Similar interactions have been found in yeast and animal systems. For example, SKP1 from *Saccharomyces cerevisiae* forms a complex with RAVE, a regulator of (H)-ATPase (V-ATPase) assembly (Seol et al., 2001). In addition, the yeast SKP1 protein forms a complex called mitogen-binding factor 3 and forms a complex with a repressor of the G2 allele of the SKP1-SGT1 protein (Stemmann et al., 2002; Willhoft et al., 2017).

The results of this study showed that *C. reinhardtii* up-regulated the expression of *skp1* under low nitrogen stress, indicating that the *C. reinhardtii skp1* gene responds to low nitrogen stress. Silencing of *C. reinhardtii skp1* by RNA interference technology reduced the expression of the *skp1* gene, resulting in a decrease in the lipid content of algal cells, which in turn was accelerated by low nitrogen stress (**Figures 2A, C**). In contrast, overexpression of *skp1* promoted lipid accumulation in algal cell (**Figures 2B, C**). This showed that *skp1* positively regulates lipid accumulation in *C. reinhardtii* cells. We also examined the expression of critical genes in *C. reinhardtii*’s lipid metabolism pathway, such as *dgat1*, *dgtt1*, *pap2* and *pdat3*, as well as *acc1*, *pepc1* and *cis* which may affect the direction of photosynthetic carbon flow. It was found that the expression of *pap2* and *pdat* genes did not change significantly in the *skp1* RNAi recombinant strain (**Figure 3C**). However, the expression of *dgad1* and *dgtt1*, key genes related to lipid synthesis, was significantly reduced. The expression of the *acc1* gene (in the direction of fatty acid synthesis), which affects the allocation of photosynthetic carbon flow, was significantly decreased, while the expression of the *cis* and *pepc1* genes (in the direction of the tricarboxylic acid cycle), which also affect the allocation of photosynthetic carbon flow, was significantly increased, resulting in a decrease in lipid content in algal cells. This indicates that SKP1 may regulate the lipid content of algal cells by modulating the expression of the above-mentioned related genes, through two aspects: influencing the distribution direction of photosynthetic carbon flow and reducing lipid synthesis (Deng et al., 2011; Deng et al., 2013b). Examination of *skp1* RNAi interfering algal strains and *skp1* overexpressing algal strains under different abiotic stresses showed that *skp1* RNAi recombinant algal strains grew better under sorbol and NaCl stress, and not as well as the control under 37°C stress (**Figure 5**, **Figures 6A, B**); whereas *skp1* overexpressing algal strains grew weaker than the control under sorbol and NaCl stress, and stronger than the control under 37°C stress (**Figure 5**, **Figures 6A, B**). It was concluded that the SCF (SKP1-cullin-F-box) complex may regulate cellular responses to osmolarity (sorbol), salt and high temperature stress through degradation of target proteins, which manifested as a negative regulation of osmolarity and salt stress and a positive regulation of high temperature stress.

In conclusion, the present study provides evidence for the involvement of SKP1 in the regulation of lipid metabolism in *C. reinhardtii.Skp1* increased its expression level in response to low nitrogen stress, which promoted lipid accumulation in *C. reinhardtii*. *skp1* negatively regulates osmolarity and salt stress, and positively regulates high temperature stress. These results illustrate the functional diversity of *skp1* in *Chlamydomonas*. This functional diversity is likely to result from the different F-box subunits bound by *skp1*. Although the F-box protein in the SCF complex controls which target proteins are bound and consequently which regulatory pathways are influenced. However, changes in SKP1 RNA (protein) levels may impact the SCF complex’s stability and hence its capacity to bind target proteins. In the follow-up study, the F-box proteins in the *C. reinhardtii* SCF (SKP1-cullin-F-box) complex will be screened by yeast two-hybridization, bimolecular fluorescence analysis and other experiments, and the substrate protein will be screened by F-box. The roles of the corresponding F-box proteins and their substrates in lipid regulation and abiotic stress response will be determined.

## Conclusions

The present study provides evidence for the involvement of SKP1 in the regulation of lipid metabolism in *C. reinhardtii*, and negatively regulates osmolarity and salt stress, and positively regulates high temperature stress. These results illustrate the functional diversity of *skp1* in *Chlamydomonas*. This study provides an important complement for lipid metabolism and abiotic stress regulation in microalgae.

## Data Availability

The datasets presented in this study can be found in online repositories. The names of the repository/repositories and accession number(s) can be found in the article/**Supplementary Material.**
