# Supplementary material for: Effects of the skp1 gene of the SCF complex on lipid metabolism and response to abiotic stress in Chlamydomonas reinhardtii
Source: Front Plant Sci. 2025 Mar 17;16:1527439. doi: 10.3389/fpls.2025.1527439 (PMC11955966; doi:10.3389/fpls.2025.1527439)
Supplement: Supplementary file 1 [file DataSheet1.pdf]

Supplementary Table 1. Origin, taxonomy and Genbank accession number of the SKP1 homologs used in the present study.

Species	Taxon	Gene ID	Note
Dictyostelium discoideum	[Protists]Mycetozoa; Dictyosteliida	U73686.1:1061-1157,1312-1703	
Physarum polycephalum	[Protists]Mycetozoa; Myxogastria	AY050559.1:1-498	
Marmota monax	Metazoa;Chordata;Mammalia	XM046463560.2:81-572	
Mus musculus	Metazoa;Chordata; Mammalia	AF083214.1:49-540	
Homo sapiens	Metazoa;Chordata; Mammalia	U33760.1:94-585	SKP1
Xenopus laevis	Metazoa;Chordata; Amphibia	AF176352.1:1-492	SKP1
Schistosoma haematobium	Metazoa;Platyhelminthes	XM012940904.3:39-530	SKP1
Strongylocentrotus purpuratus	Metazoa;Echinodermata	XM030979679.1:129-617	
Bombyx mori	Metazoa;Arthropoda; Hexapoda	NM001047053.1:301-789	
Bombus impatiens	Metazoa;Arthropoda;Insecta	XM003490702.4:276-764	
Anopheles arabiensis	Metazoa; Arthropoda; Hexapoda	XM040313378.1:195-683	
Drosophila melanogaster	Metazoa;Arthropoda; Hexapoda	AF220066.1:2795-3283	skp1A
Cooperia oncophora	Metazoa;Nematoda	MU966797.1:31760-32293	
Trichostrongylus colubriformis	Metazoa;Nematoda;Strongylida	WIXE01024945.1:222651-223157	
Ancylostoma duodenale	Metazoa;Nematoda	KN736441.1:1228-1734	
Schizosaccharomyces pombe	Fungi;Ascomycota;Schizosaccharomycetes	AB027472.1:378-398,444-811,886-933,987-1035	
Microsporum canis	Fungi; Ascomycota; Pezizomycotina	AF408428.1:205-268,321-406,467-637,685-854,925-931	
Kluyveromyces lactis	Fungi;Ascomycota;Saccharomycotina	AF012338.1:2155-2703	Skp1p
Saccharomyces cerevisiae	Fungi;Ascomycota;Saccharomycotina	U61764.1:61-645	
Tetradesmus obliquus	Viridiplantae;Chlorophyta;Chlorococcales	CP126217.1:368681-368743,368991-369031,369389-369458,369994-370190,370557-370644,370720-370731	
Scenedesmus sp.	Viridiplantae;Chlorophyta;	JABVCE010000009.1:1179492-1179554,1179706-1179746,1179996-1180065,1180277-1180473,1180584-1180671,1180767-1180775	
Chlamydomonas reinhardtii	Viridiplantae;Chlorophyta	XM001690912.2:157-630	
Physcomitrium patens	Viridiplantae;Bryophyta	XM024501970.1:217-693	
Marchantia polymorpha	Viridiplantae;Bryophyta	AP019868.1:287833-288135, 289260-289433	

Pinus taeda	Viridiplantae; Spermatophyta;Coniferales	HE716981.1:73-549	SKP1
Ceratopteris richardii	Viridiplantae; Filicophyta	CM035411.1:19893758-19894063 ,19895039-19895212	
Solanum lycopersicum	Viridiplantae;Spermatophyta;eudicots	XM004250675.5:193-660	
Nicotiana clevelandii	Viridiplantae;Spermatophyta;eudicots	AF070967.1:37-498	
Antirrhinum majus	Viridiplantae;Spermatophyta;eudicots	Y14856.1:1-486	
Glycine max	Viridiplantae;Spermatophyta;eudicots	XM003517112.5:167-634	
Medicago sativa	Viridiplantae;Spermatophyta;eudicots	AF135596.1:1-462	
Arabidopsis thaliana	Viridiplantae;Spermatophyta;eudicots	NM106245.5:197-679	ASK1
		NM128129.2:76-567	ASK3
		NM101868.5:119-610	ASK4
		NM119612.2:137-595	ASK12
		NM126368.2:15-464	ASK14
Brassica napus	Viridiplantae;Spermatophyta;eudicots	AF274864.1:1184 -1663	
Zea mays	Viridiplantae;Spermatophyta;monocots	NM001320553.1:10-687	
Sorghum bicolor	Viridiplantae;Spermatophyta;monocots	XM002453072.2:162-671	
Triticum dicoccoides	Viridiplantae;Spermatophyta;monocots	XM037593623.1:123-647	
Oryza sativa Japonica Group	Viridiplantae;Spermatophyta;monocots	AP001389.1:69010..69510	

Supplementary Table 2. Primers used in this study

**Genes**	**Primer nucleotide sequence (5'→3')**	**Gene ID in Phytozome**
skp1 RNAi	*TCGATTAAGCACGGGTCTGA*<br>*CAAATCCTCCCTACCGCCAC*	*Cre12.g501200*
skp1 full CDS	*CGCTCTACACAAATCGCAAC*<br>*ATCAGACCCGTGCTTAATCG*	*Cre12.g501200*
skp1 overexpression	*GAAGATCTGGCCACCAAGGTGAAGCTTA*<br>*GGACTAGTTTAATCGAAAGCCCACTGGT*	*Cre12.g501200*
skp1 sub location	*GAAGATCTGGCCACCAAGGTGAAGCTTA*<br>*GGACTAGTATCGAAAGCCCACTGGTTCT*	*Cre12.g501200*
pepc1(Phosphoenolpyruvate carboxylase)	*GTGCTGCACCAGTGCCTCA*<br>*CTGTCGCAGCAGGTCCAGCAG*	*Cre16.g673852*
cis(Citrate synthase)	*GACGCGCACAGCGGCGTGCT*<br>*CCTCCCTCCTTCATGTGTGT*	*Cre12.g514750*
pap2(type-2 phosphatidic acid phosphatase)	*GCGTGTTTGCCTACTTCCTC*<br>*CACTACTCGCGCCGTACAT*	*Cre05.g240000*
pdat(Phospholipid diacylglycerol acyltransferase)	*ATGTGTGAGGCTGAGGCAGT*<br>*TCAGCTGATGACCAGCGGTCG*	*Cre02.g106400*
dgat1(Diacylglycerol O-acyltransferase, Type 1)	*GCTGGAGAGGTACCCTGAGA*<br>*CAGTAGCAGCTCGTGGAACA*	*Cre01.g045903*
dgtt1(Diacylglycerol O-acyltransferase, Type 2)	*CCACGCTGGCTTCAAACTTC*<br>*CTTGACACCCTTGCCGTACT*	*Cre12.g557750*
acc1(Carboxyltransferase)	*GCCCATCATCTCGGTGGTCA*<br>*AACTCTTGGAACTGGCCCAG*	*Cre12.g519100*
Maa7IR/XIR	*ATCGATACCGTCGACCTCGAG*<br>*CGCGGTGAGCCACCGGAACGG*	*GenBank: AY710294.1*
18S rRNA	*TCAACTTTCGATGGTAGGATAGTG*<br>*CCGTGTCAGGATTGGGTAATTT*	

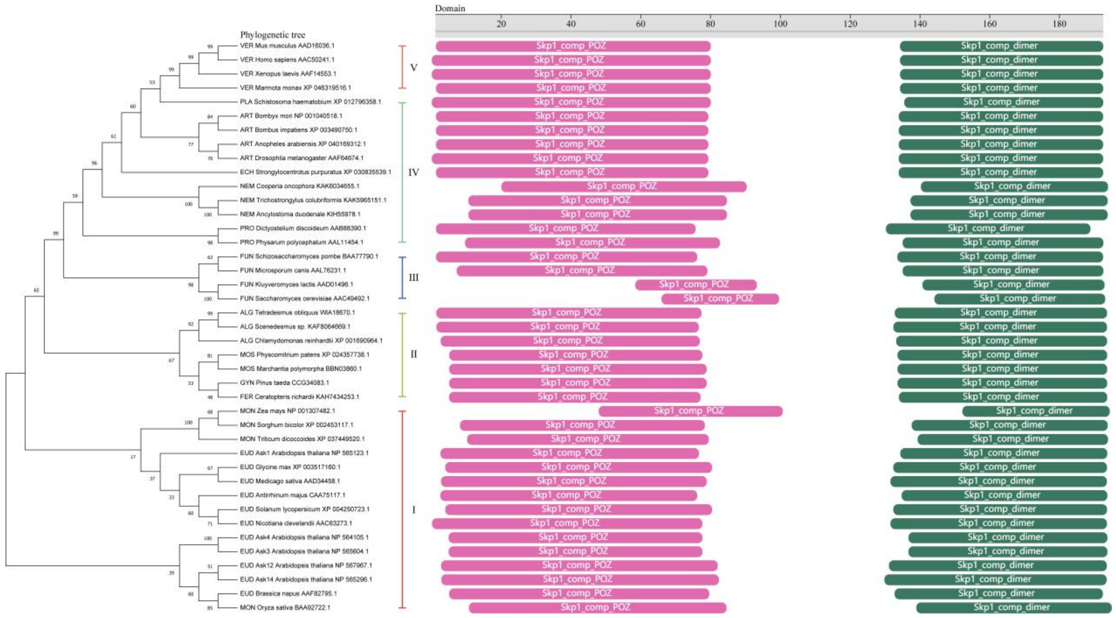

A B

Supplementary Fig.1. Phylogenetic relationship of SKP1s and their conserved domains analysis.

(A)Phylogenetic relationship of SKP1 from vertebrates, arthropods, platyhelminthes, echinoderms, nematodes, protists, green algae, mosses, ferns, fungi, gymnosperms, monocots, and eudicots. A Neighbor-Joining (NJ) tree was constructed using MEGA11, based on the alignment of SKP1-like protein sequences. Bootstrap analysis was performed with 1000 replicates. The numbers are bootstrap values based on 1000 iterations. (B) The domain of each SKP1 protein was predicted through Interproscan online (http://www.ebi.ac.uk/InterProScan). IPR0166073 represents SKP1 component, POZ domain, IPR016072 represents SKP1 component, dimerisation domain. The taxonomic categories for these organisms were abbreviated as follows: PRO, protists; NEM, nematodes; ART, arthropods; PLA, platyhelminthes; ECH, echinoderms; VER, vertebrates; FUN, fungi; ALG, green algae; MOS, mosses; FER, ferns; GYN, gymnosperms; EUD, eudicots; and MON, monocots

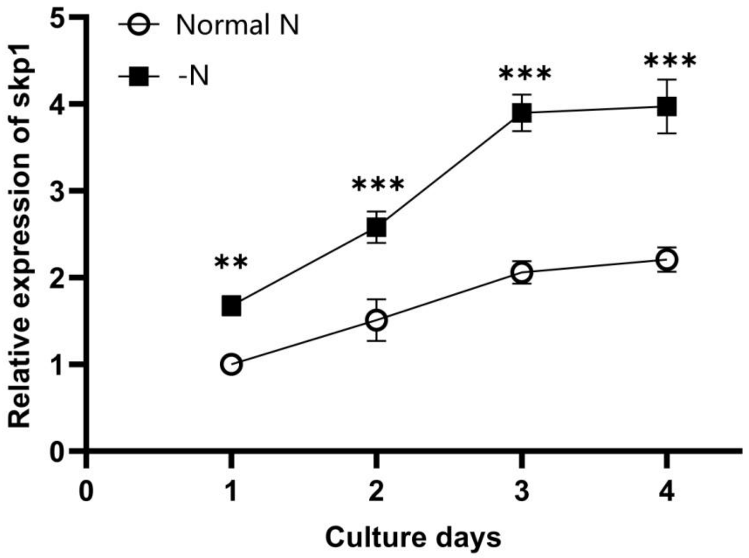

Supplementary Fig. 2. The skp1 mRNA level of C. reinhardtii CC425 on normal and N deficient medium.

*Normal N, the skp1 mRNA level of C. reinhardtii CC425 on TAP medium with normal nitrogen; -N, the skp1 mRNA level of C. reinhardtii CC425 on TAP medium with nitrogen deficiency. **, P<0.01，***,P<0.001.*

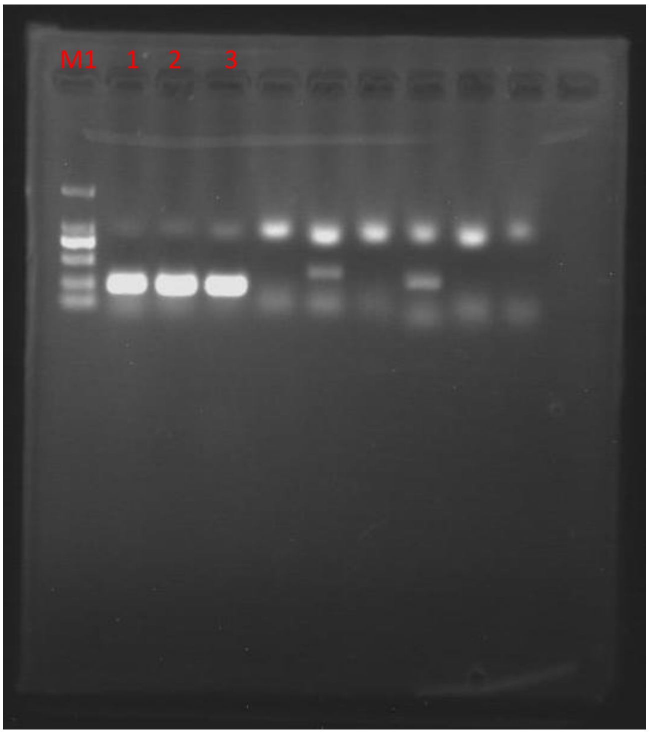

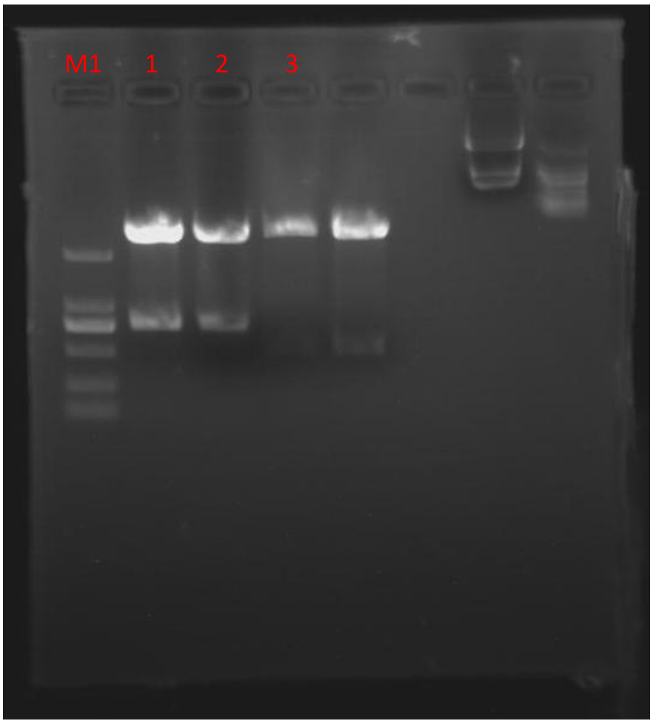

Supplementary Fig.3. Original electrophoresis graph corresponding to Fig. 2 in the main text.

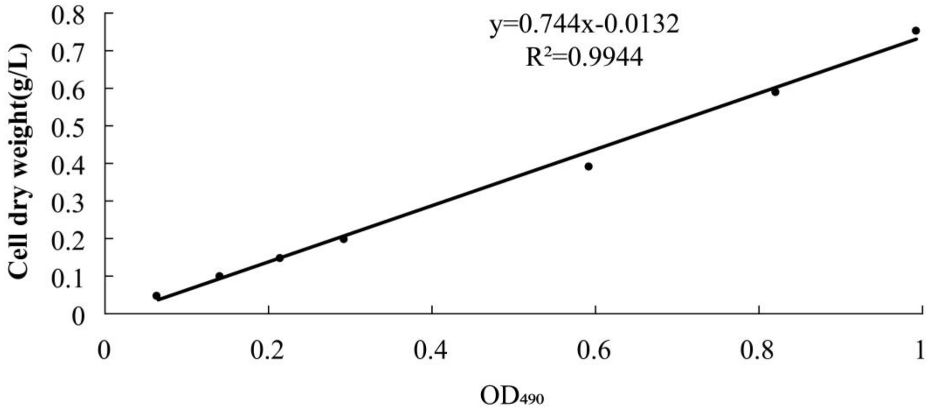

Supplementary Fig. 4. The correlation between biomass (Cell dry weight g/L) and the optical density OD490

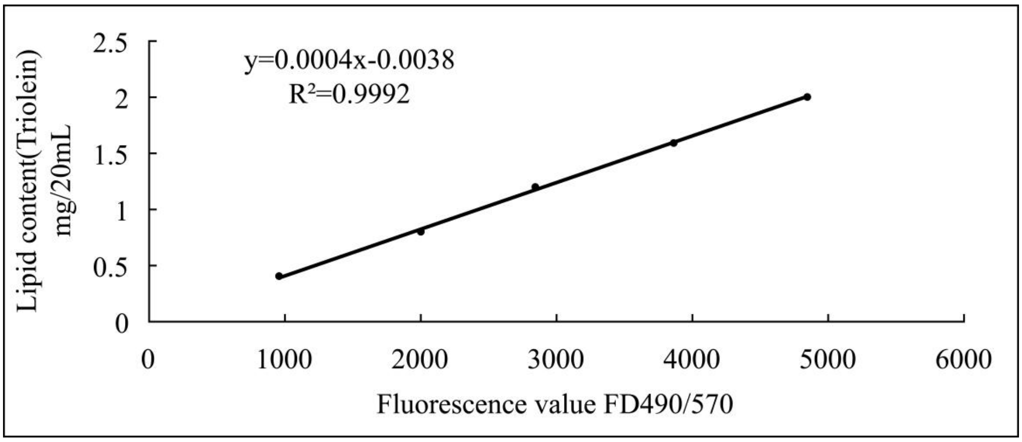

Supplementary Fig. 5. The correlation between lipid concentration (Triolein mg/20mL) and the fluorescence value FD470/570

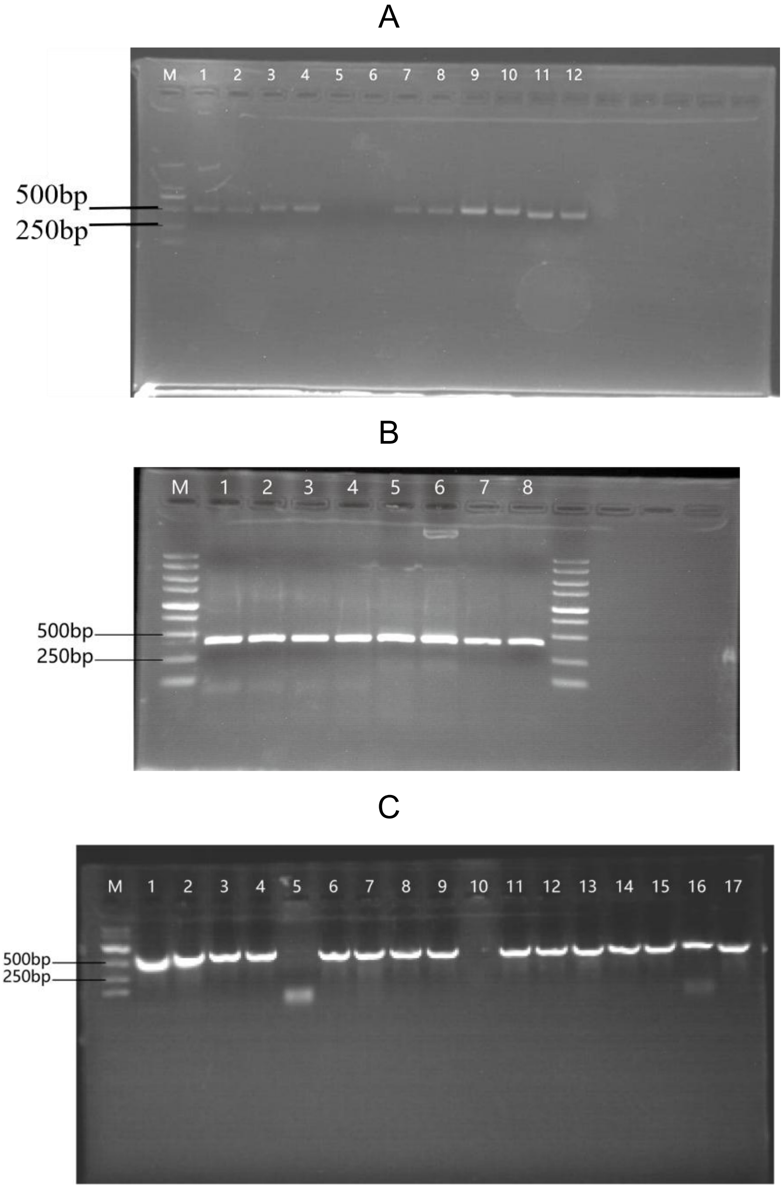

Supplementary Fig. 6. PCR identification of the recombinant plasmid pCAM-skp1 (A) and of recombinant Chlamydomonas strains with skp1 RNAi (B) and skp1 overexpression (C).

A, PCR amplification results of the recombinant overexpression vector pCAM-skp1. M: DL2000 DNA marker; 1-12: the PCR product (510 bp) of the recombinant plasmid pCAM-skp1 with full-length CDS cloned into the Bgl II/Spe I locus of pCAMBIA1302.

B, Partial PCR amplification results of the recombinant Chlamydomonas strains transformed with the skp1 RNAi vector pMaa7IR/skp1IR. M: DL10000 DNA marker; 1-8: the PCR product (430 bp) of the recombinant Chlamydomonas strain transformed by pMaa7IR/skp1IR with primers designed within the promoter region of the pMaa7IR/XIR vector.

C, Partial PCR amplification results of the recombinant Chlamydomonas strains transformed with the skp1 overexpression vector of pCAM-skp1. M: DL2000 DNA marker; 1-17: the PCR product (510 bp) of the recombinant Chlamydomonas strain transformed by pCAM-skp1.
